# Sylvian fissure angle development on fetal MRI: 22–38 gestational weeks

**DOI:** 10.3389/fnins.2025.1686398

**Published:** 2026-01-05

**Authors:** Yan Song, Siyan Lu, Hongyu Yin, Xi Chen, Yuchen Liu, Min Kang, Ran Li

**Affiliations:** 1 Department of Radiology, Sichuan Provincial Woman's and Children's Hospital, The Affiliated Women's and Children's Hospital of Chengdu Medical College, Chengdu, Sichuan, China; 2 Department of Ultrasound, Sichuan Provincial Woman's and Children's Hospital, The Affiliated Women's and Children's Hospital of Chengdu Medical College, Chengdu, Sichuan, China; 3 GE HealthCare MR Research, Beijing, China

**Keywords:** fetal brain, growth curve, MRI, normal development, reference range, Sylvian fissure angle

## Abstract

**Objective:**

The objective of the study was to quantify Sylvian fissure angle (SFA) development via fetal magnetic resonance imaging (MRI) and establish the reference range and growth curve during normal pregnancy.

**Materials and methods:**

This retrospective cross-sectional study collected 324 singleton fetuses at 22–38 weeks of gestation (GA), without abnormal signs on MRI or chromosomal defects, from January 2022 to December 2023. Six SFAs were defined and measured: Sylvian fissure plateau angle (SFPA), Sylvian fissure anterior rotation angle (SFARA), and Sylvian fissure posterior rotation angle (SFPRA) in the transthalamic axial plane, and SFPA, Sylvian fissure superior rotation angle (SFSRA), and Sylvian fissure inferior rotation angle (SFIRA) in the third ventricular coronal plane. Then, the correlation between the SFAs and GA was analyzed, and the growth curve model was used. Retrospective cortical malformation cases validated the model. The intraclass correlation coefficient (ICC) was used to evaluate interobserver variability of the SFA measurements.

**Results:**

ICC for SFA measurements demonstrated good reliability (0.772–0.976). The bilateral SFARA and SFSRA were significantly positively associated with GA (r = 0.914, 0.926, 0.842, 0.836, left to right, all *p <* 0.001). The bilateral SFPRA, SFIRA, and SFPA (both axial and coronal views) were negatively associated with GA (r value range: −0.601 to −0.145, all *p <* 0.001). For each SFA and GA, the quadratic curve has the best goodness of fit. The model detected cortical malformation (genetically confirmed). Except for the SFARA, all SFAs were statistically different between the two sides.

**Conclusion:**

The dynamic development of the fetal SFA can be well evaluated using MRI. The SFA’s reference range will enhance the accuracy of fetal brain assessment and offer a quantitative reference framework for identifying deviations suggestive of cortical malformations.

## 1 Introduction

Malformations of cortical development (MCD), such as lissencephaly, polymicrogyria, and heterotopias, are severe neurodevelopmental disorders associated with devastating outcomes, including intellectual disability, epilepsy, autism, and early death (Shi et al., 2021; Enokizono et al., 2019; Wang et al., 2024). Early prenatal detection of these disorders is crucial for accurate prognosis, informed parental counseling, and timely clinical management. The Sylvian fissure, a key cortical development marker, is among the earliest visible structures, detectable by imaging at 15–17 gestational weeks (Zeng et al., 2023). Aberrant or delayed development of the Sylvian fissure is frequently associated with MCD and thus may serve as a valuable indicator of abnormal cortical maturation (Gindes et al., 2015; Lerman-Sagie et al., 2021).

Previous studies of the Sylvian fissure used ultrasound examination. It quantitatively measured morphological parameters of the fetal Sylvian fissure (including length, depth, angle, and surface area), thereby establishing ultrasound reference ranges for normal fetal Sylvian fissure development and plotting its dynamic growth curves. However, previous studies have focused primarily on subjective assessments or single-plane measurements, lacking systematic, reproducible, and quantitative standards across gestational ages (Zeng et al., 2023; Gindes et al., 2015; Quarello et al., 2008; Poon et al., 2019; Chen et al., 2017; Mammadova et al., 2024). Furthermore, ultrasound imaging has inherent limitations: first, its relatively low spatial resolution hinders clear visualization of subtle cerebral structures, and second, skull-induced acoustic shadowing, oligohydramnios, and maternal obesity often degrade intracranial anatomical imaging quality, reducing measurement accuracy (Quarello et al., 2008). Magnetic resonance imaging (MRI) offers superior soft-tissue contrast, multiplanar acquisition, and improved reproducibility, making it a promising modality for detailed evaluation of fetal brain sulcation (Quarello et al., 2008).

Previous ultrasound studies have observed gestational age-dependent changes in fetal Sylvian fissure morphology. For example, the Sylvian fissure expands in both the anterior–posterior and superior–inferior directions until it forms the closed Sylvian fissure (Gindes et al., 2015; Poon et al., 2019). In our study, we selected the axial and coronal planes to measure Sylvian fissure angles in accordance with the growth pattern in which the Sylvian fissure extends in both the anterior–posterior and superior–inferior directions.

In this study, we introduced a novel set of quantitative Sylvian fissure angles (SFAs), including the Sylvian fissure plateau angle (SFPA), Sylvian fissure anterior rotation angle (SFARA), and Sylvian fissure posterior rotation angle (SFPRA) on axial imaging and Sylvian fissure superior rotation angle (SFSRA) and Sylvian fissure inferior rotation angle (SFIRA) on coronal imaging. These measurements provide a multidimensional description of fissure orientation and maturation. By establishing gestational age-specific normative ranges for each SFA, we aimed to provide objective, reproducible parameters to support early detection of atypical cortical development. We hypothesized that Sylvian fissure angles exhibit consistent, gestational age-dependent patterns of change during normal fetal development, and these changes follow a predictable trajectory that can be modeled mathematically.

## 2 Materials and methods

### 2.1 Subjects

Patients who underwent fetal head MRI in our institution from January 2022 to December 2023 were retrospectively reviewed. Among these patients, the study population was selected based on the following inclusion criteria: fetuses at 22–38 weeks of gestation (GA), without abnormal signs on MRI or chromosomal defects. The exclusion criteria included images with substantial artifacts or non-standard scanning sections, which cannot support the subsequent measurement of Sylvian fissure angles. The included fetuses were divided into several groups according to their GA. To avoid bias and make the sample size of each GA group balanced, we also conducted random sampling screening in groups with more samples. Quota sampling was used in this study, with approximately 20 samples allocated to each gestational age group.

### 2.2 Image acquisition

The imaging protocol included standard MRI examinations of the fetal head performed on a 1.5 T Signa Excite HDx scanner (GE Healthcare, Waukesha, WI) with an 8-channel abdominal coil. Pregnant women were placed in a comfortable position, usually supine or left lateral decubitus, with free breathing and no sedatives administered. They were positioned in a feet-first orientation. Imaging was required to cover three mutually perpendicular anatomical planes of the fetal head, namely axial, coronal, and sagittal planes. Due to the unpredictability of fetal movement, real-time three-plane localization was essential during positioning, with continuous adjustments made to the scanning alignment. The center of localization was aligned with the coil center and the fetal region of interest, with secondary localization performed if necessary. The angles were measured on the single-shot fast spin echo (SSFSF) sequence in coronal and axial positions. The acquisition parameters were as follows: slice thickness = 3.0 mm, TR = 1800 ms, TE = 96 ms, and field of view = 320 × 320 mm^2^.

### 2.3 Sylvian fissure angle

For the evaluation of the normal development of the Sylvian fissures, after referring to previous ultrasound-related research (Gindes et al., 2015; Poon et al., 2019), different Sylvian fissure angles were defined in this study. The definitions were as follows:

Sylvian fissure plateau angle (SFPA): The angle between the brain midline (BM) and the Sylvian fissure width line is bilaterally measured on axial imaging.
Sylvian fissure anterior rotation angle (SFARA): The angle between the BM and the anterior boundary of the Sylvian fissure is bilaterally measured on axial imaging.
Sylvian fissure posterior rotation angle (SFPRA): The angle between the BM and the posterior boundary of the Sylvian fissure is bilaterally measured on axial imaging.
Sylvian fissure superior rotation angle (SFSRA): The angle between the BM and the superior boundary of the Sylvian fissure is bilaterally measured on coronal imaging.
Sylvian fissure inferior rotation angle (SFIRA): The angle between the BM and the inferior boundary of the Sylvian fissure is bilaterally measured on coronal imaging.

**Figure 1:**
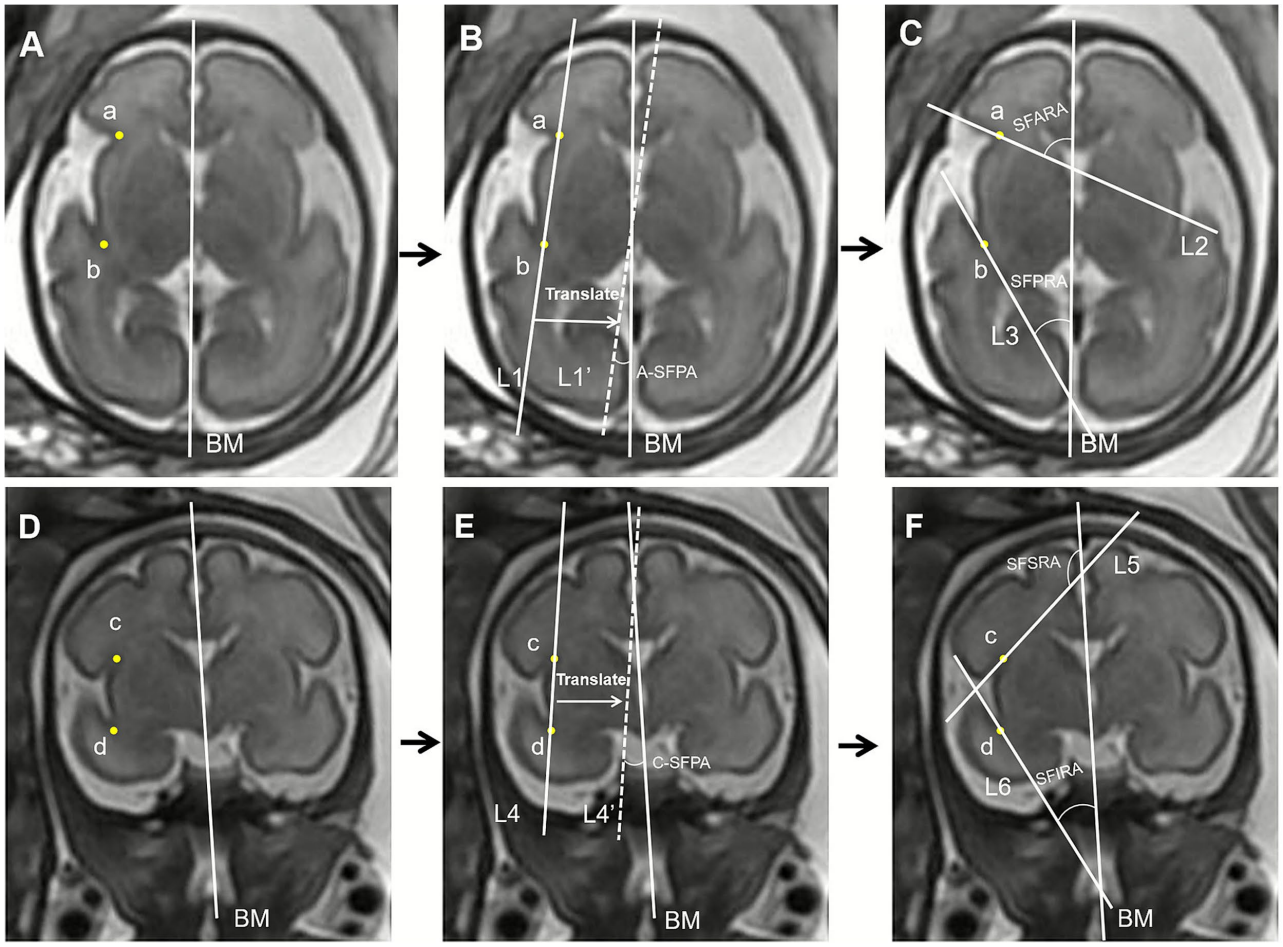
Method for measurements of the Sylvian fissure angles (SFAs) on axial view and coronal view. **(A)** On the axial view, draw the brain midline (BM) through the thalamic plane. Locate the intersection of the frontal lobe and insula as point a, and the intersection of the temporal lobe and insula as point b. **(B)** Draw a straight line, L1, connecting points a and b. Define the angle between L1 (or its parallel, L1’) and the BM as the axial Sylvian fissure plateau angle (A-SFPA). **(C)** Draw a tangential line L2 to the frontal lobe through point a; the ventral angle between L2 and the BM is defined as the Sylvian fissure anterior rotation angle (SFARA). Draw a tangential line L3 to the temporal lobe through point b; the ventral angle between L3 and BM is defined as the Sylvian fissure posterior rotation angle (SFPRA). **(D)** On the coronal view, draw the BM through the third ventricle plane. Locate the intersection of the parietal lobe and insula as point c, and the intersection of the temporal lobe and insula as point d. **(E)** Draw a straight line, L4, connecting points c and d. Define the angle between L4 (or its parallel, L4’) and the BM as the coronal Sylvian fissure plateau angle (C-SFPA). **(F)** Draw a tangential line L5 to the parietal lobe through point c; the anterior angle between L5 and the BM is defined as the Sylvian fissure superior rotation angle (SFSRA). Draw a tangential line L6 to the temporal lobe through point d; the anterior angle between L6 and the BM is defined as the Sylvian fissure inferior rotation angle (SFIRA).

The methods for measuring the Sylvian fissure angles (SFAs) on axial and coronal views are illustrated in Figure 1.

All SFAs were measured by two examiners separately (R. L. and Y. S.). For intra-observer reliability assessment, Examiner 1 first performed bilateral SFA measurements on all images of the full cohort. After a 2-week interval (to avoid recall bias), a 50% subset of the cohort was selected via stratified random sampling based on GA groups (consistent with the full cohort’s GA distribution) for repeated measurements by Examiner 1. For interobserver reliability, Examiner 2 (blinded to Examiner 1’s results and participants’ clinical data) independently measured the same 50% stratified random subset. The consistency between subset measurements and full-cohort data was pre-validated (Pearson’s correlation coefficient r = 0.92, *p <* 0.001).

### 2.4 Data analysis

Normally distributed continuous variables were expressed as means ± standard deviation, while non-normally distributed variables were presented as median and interquartile ranges (IQRs). The correlation between variables was evaluated using Pearson’s correlation coefficient for normally distributed continuous variables and Spearman’s rank-order correlation for non-normally distributed variables. The Wilcoxon signed-rank test was used for the comparison of the SFA of the two sides. Statistical significance was defined as a two-tailed *p*-value of < 0.05.

The data analysis was initiated by constructing fractional polynomial regression models to characterize the relationship between mean Sylvian fissure angle measurements and GA. The modeling approach incorporated several key steps: First, a normal distribution of SFAs was assumed at each GA, which was verified using Shapiro–Wilk W tests while allowing both the mean and standard deviation to vary smoothly with advancing gestation. For mean value modeling, a stepwise fractional polynomial regression procedure was used, evaluating polynomial terms from the fourth to the first order. Each candidate model was rigorously assessed based on three criteria: (1) deviance differences between nested models, (2) visual inspection of curve fit quality (with particular attention to gestational age extremes), and (3) the coefficient of determination (R^2^). Model selection prioritized the most parsimonious specification that simultaneously minimized deviance while maintaining optimal curve characteristics. Finally, each model’s goodness of fit was quantified using the R^2^ statistic to ensure an adequate explanation of observed data variability. In addition, the goodness of fit was evaluated using quantile–quantile (Q–Q) plots and the Kolmogorov–Smirnov test for residual normality.

For model validation, multiple retrospective cortical malformation cases were enrolled, with genetic testing confirming diagnoses.

Inter-reader variability was evaluated using the intra-class correlation coefficient (ICC) for each feature, based on a two-way random-effects model with a single rater/measurement and absolute agreement; an ICC above 0.75 was considered good agreement. SPSS (Version 26, IBM, Chicago, IL, USA) and R Software (Version 4.3.1, R Foundation for Statistical Computing, Vienna, Austria) were used for data entry, statistical analysis, and graphics.

## 3 Results

### 3.1 Subjects

**Figure 2:**
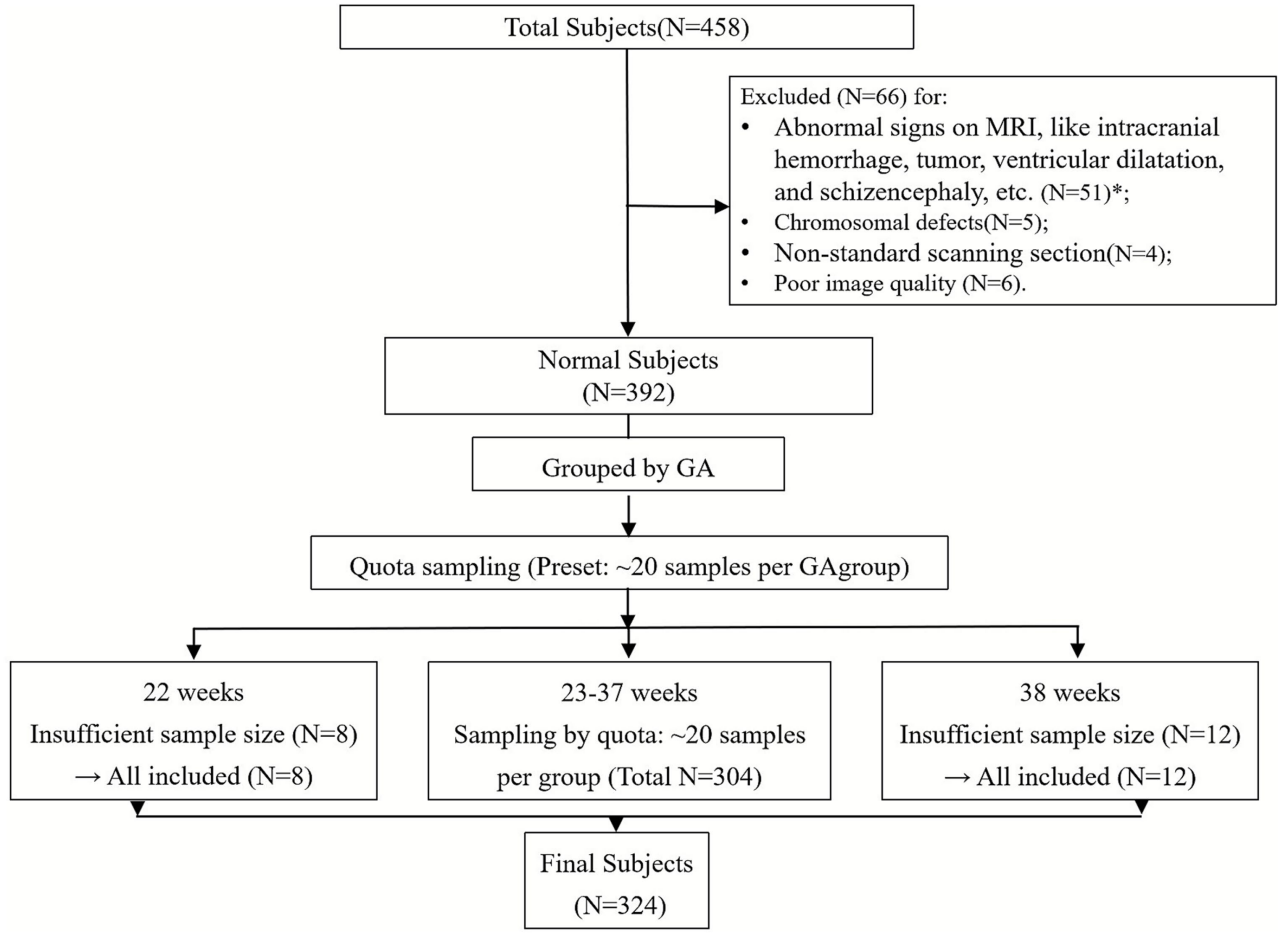
Study flowchart of the enrolled subjects. N: number, GA: gestational weeks, *: details of the excluded cases: ventriculomegaly, *N =* 18; abnormalities of the corpus callosum, *N =* 8; intracranial hemorrhage, *N =* 6; abnormalities of the posterior fossa, *N =* 6; holoprosencephaly, *N =* 1; intracranial tumors, *N =* 1; others, *N =* 11.

The participant enrollment flowchart is shown in Figure 2. A total of 458 pregnant women underwent fetal head MRI scans in our institution between January 2022 and December 2023. Following the application of the exclusion criteria, 392 fetuses were classified as normal. These fetuses were stratified by GA, and quota sampling was performed across all GA groups. All cases from the extreme gestational age groups (22 weeks as the earliest and 38 weeks as the latest) were included in the analysis due to small sample sizes, while approximately 20 cases were enrolled per intermediate group (33–37 weeks). Ultimately, 324 fetuses were included in the analysis, comprising 136 (42%) female fetuses and 188 (58%) male fetuses. Among them, 264 fetuses (81.5%) were in the cephalic position, 55 (17.0%) in the breech position, and 5 (1.5%) in the transverse lie position.

### 3.2 Reference range for SFAs at different GA

The directly measured angles for Sylvian fissure development from 22 to 38 weeks of GA are presented in Supplementary Tables S1, S2. We observed that the Sylvian fissure angles changed progressively with increasing GA. We identified a strong positive correlation between GA and bilateral SFARA values (left: r = 0.914; right: r = 0.926; both *p <* 0.001) (Table 1). Similarly, a significant positive correlation was found between GA and bilateral SFSRA values (left: r = 0.842, *p <* 0.001; right: r = 0.836, *p <* 0.001). GA exhibited moderate negative correlations between the bilateral SFPRA, SFIRA, and axial SFPA (r = −0.601 to −0.461, all *p <* 0.001). A weak negative correlation was observed between GA and the bilateral coronal SFPA (left: r = −0.145, *p <* 0.001; right: r = −0.193, *p <* 0.001).

**Table 1:** Correlation analysis between the bilateral Sylvian fissure angles and gestational age.

Parameters	*r*	*p*
L-SFARA	0.914	<0.001
L-SFPRA	−0.544	<0.001
AL-SFPA	−0.547	<0.001
L-SFSRA	0.842	<0.001
L-SFIRA	−0.601	<0.001
CL-SFPA	−0.145	<0.001
R-SFARA	0.926	<0.001
R-SFPRA	−0.600	<0.001
AR-SFPA	−0.461	<0.001
R-SFSRA	0.836	<0.001
R-SFIRA	−0.554	<0.001
CR-SFPA	−0.193	<0.001

L: left, R: right, SFARA: Sylvian fissure anterior rotation angle, SFPRA: Sylvian fissure posterior rotation angle, SFSRA: Sylvian fissure superior rotation angle, SFIRA: Sylvian fissure inferior rotation angle, AL-SFPA: left axial Sylvian fissure plateau angle, AR-SFPA: right axial Sylvian fissure plateau angle, CL-SFPA: left coronal Sylvian fissure plateau angle, CR-SFPA: right coronal Sylvian fissure plateau angle.

### 3.3 Reference equation of SFA based on GA

For each parameter, polynomial growth curves relating angle (in degrees) to GA were derived using data analysis. Although we considered polynomial equations up to the fourth order, quadratic regression equations were the best fit for all growth parameters. Table 2 shows fitting equations for each SFA, along with the relevant parameters of the goodness-of-fit test and residual analysis. GA-based models of SFA were reliable (all *p <* 0.05). The residuals exhibited no signs of skewness, and their kurtosis values were approximately equal to the expected value of 0. Tables 3, 4 show the values of the centile curves (5th, 50th, and 95th) for SFAs calculated based on the fitted equations. Tables 3, 4 present the values of the 5th, 50th, and 95th percentile curves for the SFAs across different gestational ages, calculated based on the fitted equations.

**Table 2:** Reference equation of bilateral Sylvian fissure angles based on gestational age.

Parameters	Reference equations	R^2^	*p*	Residuals				
				Mean	SD	Skew	Kurtosis	*p*
L-SFARA	Y = 6.241 + (−2.389) x + 0.147 x^2^	0.830	<0.001	0	1.895	0.148	0.053	0.200
R-SFARA	Y = 24.494 + (−3.704) x + 0.171 x^2^	0.852	<0.001	0	1.783	−0.110	0.706	0.200
L-SFPRA	Y = 438. 756 + (−24.767) x + 0.369 x^2^	0.418	<0.001	0	3.905	−0.061	−0.806	0.057
R-SFPRA	Y = 522.315 + (−29.730) x + 0.440 x^2^	0.485	<0.001	0	3.669	−0.037	−1.001	0.072
AL-SFPA	Y = 75.379 + (−3.273) x + 0.042 x^2^	0.309	<0.001	0	3.999	−0.004	−0.689	0.070
AR-SFPA	Y = 70.128 + (−3.609) x + 0.039 x^2^	0.237	<0.001	0	4.218	−0.026	−0.619	0.072
L-SFSRA	Y = −398.439 + 29.627 x + (−0.416) x^2^	0.763	<0.001	0	2.449	−0.425	1.097	0.060
R-SFSRA	Y = −365.325 + 27.151 x + (−0.373) x^2^	0.759	<0.001	0	2.678	−0.344	−0.345	0.096
L-SFIRA	Y = 385.173 + (−20.763) x + 0.304 x^2^	0.520	<0.001	0	3.604	−0.101	−0.665	0.058
R-SFIRA	Y = 388.776 + (−21.087) x + 0.311 x^2^	0.474	<0.001	0	3.725	−0.047	−0.843	0.050
CL-SFPA	Y = 11.303 + 0.093 x + (−0.004) x^2^	0.021	0.035	0	4.739	−0.130	−1.163	0.090
CR-SFPA	Y = 5.056 + 0.627 x + (−0.014) x^2^	0.037	0.002	0	4.702	−0.051	−1.142	0.075

L: left, R: right, SFARA: Sylvian fissure anterior rotation angle, SFPRA: Sylvian fissure posterior rotation angle, SFSRA: Sylvian fissure superior rotation angle, SFIRA: Sylvian fissure inferior rotation angle, AL-SFPA: left axial Sylvian fissure plateau angle, AR-SFPA: right axial Sylvian fissure plateau angle, CL-SFPA: left coronal Sylvian fissure plateau angle, CR-SFPA: right coronal Sylvian fissure plateau angle.

**Table 3:** Median, 5th, and 95th smoothed percentiles: reference values for fetal axial Sylvian fissure angles stratified by gestational age.

GA	L-SFARA			R-SFARA			L-SFPRA			R-SFPRA			AL-SFPA			AR-SFPA		
	Median	5th	95th	Median	5th	95th	Median	5th	95th	Median	5th	95th	Median	5th	95th	Median	5th	95th
22	26.3	22.9	27.2	27.2	24.1	28.1	67.3	66.5	72.1	78.2	77.1	84.1	23.0	22.9	23.6	21.4	21.2	22.1
23	28.7	27.2	30.7	29.5	28.0	31.4	62.7	59.1	65.6	72.6	68.3	76.1	22.2	21.6	22.7	20.6	20.1	21.1
24	34.5	31.7	35.5	35.0	32.3	36.0	53.1	51.7	57.6	61.0	59.3	66.4	20.6	20.3	21.3	19.1	18.9	19.8
25	38.7	36.5	41.0	39.1	36.9	41.4	47.3	44.5	50.3	53.9	50.5	57.6	19.5	19.0	20.1	18.1	17.6	18.6
26	43.7	41.6	46.1	44.0	41.9	47.1	41.4	39.2	43.5	46.8	44.1	49.1	18.4	18.0	18.7	17.1	16.7	17.5
27	49.5	47.0	51.5	49.7	47.2	51.7	35.8	34.1	38.1	39.9	37.9	42.7	17.3	16.9	17.7	16.0	15.7	16.5
28	54.5	52.7	57.1	54.7	52.9	56.8	31.8	30.5	33.5	35.1	33.5	36.9	16.4	16.1	16.7	15.3	15.0	15.6
29	61.8	58.7	62.4	62.1	58.9	63.2	27.1	26.8	28.7	29.4	29.0	31.4	15.3	15.2	15.7	14.3	14.2	14.8
30	67.6	65.2	68.9	67.9	65.5	69.3	24.3	23.8	25.4	26.0	25.4	27.3	14.6	14.4	14.9	13.6	13.5	13.9
31	73.3	71.6	75.7	73.8	72.0	76.2	22.3	21.6	22.8	23.5	22.7	24.2	13.9	13.7	14.1	13.1	12.8	13.2
32	80.9	78.5	82.7	81.6	79.1	83.5	20.5	20.2	20.9	21.3	20.9	21.9	13.2	13.1	13.4	12.4	12.3	12.6
33	87.1	85.7	90.1	88.0	86.5	91.1	19.7	19.5	19.8	20.3	20.1	20.5	12.7	12.5	12.8	12.0	11.8	12.1
34	94.6	93.1	97.8	95.9	94.3	99.8	19.5	19.5	20.2	20.0	20.0	20.2	12.2	12.1	12.3	11.6	11.4	11.7
35	102.5	100.9	105.7	104.0	102.4	107.4	20.0	19.9	20.5	20.7	20.5	21.2	11.8	11.7	11.9	11.2	11.1	11.3
36	109.8	109.0	113.9	111.7	110.8	116.0	21.2	21.0	22.1	22.1	21.9	23.1	11.5	11.4	11.5	11.0	10.9	11.0
37	118.2	117.3	121.6	120.6	119.7	124.1	23.2	23.0	24.2	24.5	24.2	25.7	11.3	11.2	11.3	10.8	10.8	10.8
38	129.1	126.0	131.3	132.1	128.8	134.6	26.8	25.7	27.3	28.8	27.4	29.4	11.1	11.1	11.1	10.7	10.7	10.7

L: left, R: right, SFARA: Sylvian fissure anterior rotation angle, SFPRA: Sylvian fissure posterior rotation angle, AL-SFPA: left axial Sylvian fissure plateau angle, AR-SFPA: right axial Sylvian fissure plateau angle.

**Table 4:** Median, 5th, and 95th smoothed percentiles: reference values for fetal coronal Sylvian fissure angles stratified by gestational age.

GA	L-SFSRA			R-SFSRA			L-SFIRA			R-SFIRA			CL-SFPA			CR-SFPA		
	Median	5th	95th	Median	5th	95th	Median	5th	95th	Median	5th	95th	Median	5th	95th	Median	5th	95th
22	56.1	46.8	57.2	54.8	46.0	56.3	72.9	72.2	76.2	73.6	72.9	77.5	11.3	11.3	11.4	12.3	12.3	12.3
23	62.1	58.3	66.9	60.6	57.0	65.2	69.0	65.9	71.5	69.7	66.6	72.2	11.3	11.3	11.3	12.3	12.3	12.3
24	74.9	69.0	76.9	72.9	67.1	74.7	60.8	59.6	64.6	61.4	60.2	65.3	11.2	11.2	11.3	12.2	12.2	12.3
25	82.9	78.7	86.8	80.5	76.5	84.3	55.8	53.4	58.4	56.4	54.0	59.0	11.2	11.1	11.2	12.2	12.1	12.2
26	91.0	87.7	95.3	88.4	85.1	92.1	50.8	48.8	52.3	51.4	49.4	53.1	11.1	11.0	11.2	12.1	12.1	12.1
27	99.1	95.7	101.5	96.2	93.0	98.6	45.8	44.4	47.9	46.5	45.1	48.5	11.0	10.9	11.0	12.0	11.9	12.0
28	104.9	103.0	107.2	102.0	100.0	104.2	42.4	41.2	43.6	43.1	42.0	44.4	10.9	10.8	10.9	11.9	11.8	11.9
29	112.1	109.3	112.7	109.1	106.3	110.4	38.2	37.9	39.7	39.1	38.8	40.8	10.7	10.7	10.8	11.7	11.7	11.7
30	116.7	114.9	117.7	113.7	111.9	114.7	35.7	35.2	36.7	36.7	36.2	37.6	10.6	10.6	10.6	11.5	11.5	11.6
31	120.4	119.4	121.8	117.5	116.5	118.9	33.8	33.1	34.3	34.9	34.3	35.4	10.5	10.4	10.5	11.3	11.2	11.4
32	124.3	123.2	125.0	121.6	120.4	122.4	31.9	31.6	32.4	33.3	33.0	33.7	10.3	10.2	10.3	11.1	11.0	11.2
33	126.6	126.1	127.4	124.1	123.6	125.1	31.1	30.8	31.2	32.6	32.4	32.7	10.1	10.1	10.2	10.9	10.7	10.9
34	128.4	128.1	128.9	126.3	125.9	127.5	30.6	30.6	30.6	32.4	32.4	32.6	10.0	9.9	10.0	10.6	10.5	10.6
35	129.4	129.3	129.6	127.8	127.5	128.1	30.8	30.7	31.0	32.8	32.7	33.2	9.8	9.7	9.8	10.3	10.1	10.3
36	129.6	129.4	129.6	128.4	128.3	128.5	31.4	31.3	32.0	33.8	33.7	34.6	9.6	9.5	9.6	10.0	9.8	10.0
37	129.0	128.5	129.1	128.3	128.1	128.4	32.8	32.6	33.5	35.5	35.3	36.4	9.4	9.3	9.4	9.6	9.5	9.7
38	127.0	126.7	127.7	127.2	127.0	128.2	35.4	34.6	35.7	38.5	37.6	39.1	9.1	9.0	9.1	9.2	9.1	9.3

L: left, R: right, SFSRA: Sylvian fissure superior rotation angle, SFIRA: Sylvian fissure inferior rotation angle, CL-SFPA: left coronal Sylvian fissure plateau angle, CR-SFPA: right coronal Sylvian fissure plateau angle.

### 3.4 Comparison of bilateral Sylvian fissure angles

**Figure 3:**
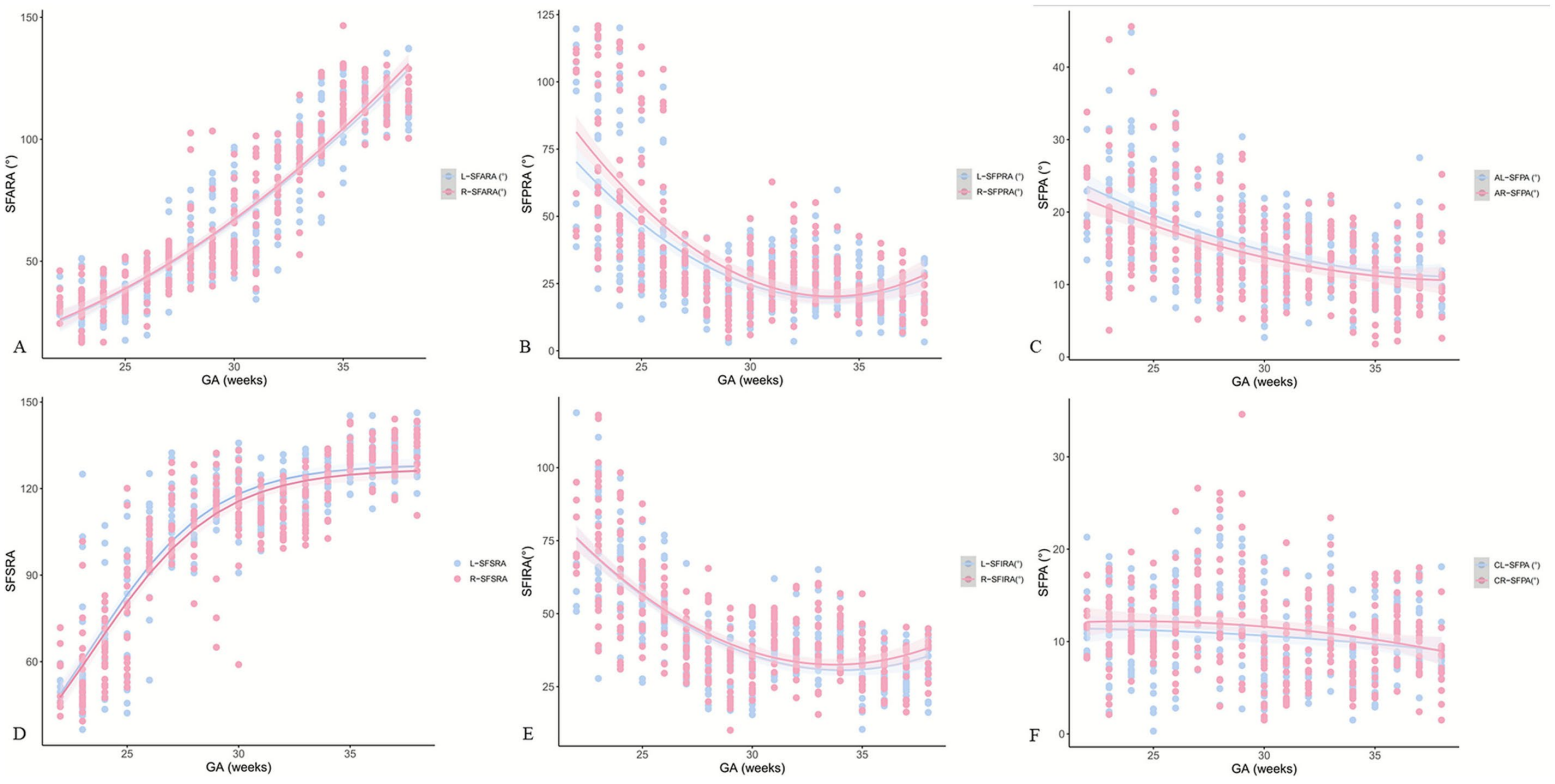
Raw data for bilateral Sylvian fissure angles (SFAs) with fitted curves. GA: gestational age, L: left, R: right, SFARA: Sylvian fissure anterior rotation angle, SFPRA: Sylvian fissure posterior rotation angle, SFSRA: Sylvian fissure superior rotation angle, SFIRA: Sylvian fissure inferior rotation angle, AL-SFPA: left axial Sylvian fissure plateau angle, AR-SFPA: right axial Sylvian fissure plateau angle, CL-SFPA: left coronal Sylvian fissure plateau angle, CR-SFPA: right coronal Sylvian fissure plateau angle.

Significant differences were observed in bilateral SFAs except the SFARA (*p* = 0.347, Table 5). Raw data for bilateral SFAs with fitted curves are presented in Figure 3. The SFPA curve in the left axial position was above the one in the right axial position. The SFSRA curve on the left side was higher than that on the right side. The SFPA curve in the right coronal position was above the one in the left coronal position. Before 32 weeks of gestation, the SFPRA curve on the right side was above the one on the left side. Before 30 weeks of gestation, the SFIRA curves on both sides were close to each other. After 30 weeks of gestation, the value of the SFIRA curve on the right side was higher than that on the left side.

**Table 5:** Difference between the left and right Sylvian fissure angles.

Parameters	*p*
L-SFARA vs. R-SFARA	0.347
L-SFPRA vs. R-SFPRA	<0.001
AL-SFPA vs. AR-SFPA	0.011
L-SFSRA vs. R-SFSRA	0.002
L-SFIRA vs. R-SFIRA	0.026
CL-SFPA vs. CL-SFPA	0.009

L: left, R: right, SFARA: Sylvian fissure anterior rotation angle, SFPRA: Sylvian fissure posterior rotation angle, SFSRA: Sylvian fissure superior rotation angle, SFIRA: Sylvian fissure inferior rotation angle, AL-SFPA: left axial Sylvian fissure plateau angle, AR-SFPA: right axial Sylvian fissure plateau angle, CL-SFPA: left coronal Sylvian fissure plateau angle, CR-SFPA: right coronal Sylvian fissure plateau angle.

### 3.5 Inter-rater and intra-rater agreement

Intra-rater and inter-rater reliability for SFA measurements were found to be good (mean ICC = 0.862, range = 0.769–0.978 and mean ICC = 0.850, range = 0.772–0.976). The ICC of the left and right SFA measurements between the two observers is listed in Table 5.

**Table 5:** Interobserver repeatability of fetal right and left Sylvian fissure angle measurements.

Parameters	Mean	95% CI
L-SFARA	0.976	0.955–0.987
L-SFPRA	0.849	0.738–0.915
AL-SFPA	0.883	0.795–0.935
L-SFSRA	0.965	0.937–0.981
L-SFIRA	0.824	0.698–0.901
CL-SFPA	0.807	0.671–0.891
R-SFARA	0.809	0.674–0.892
R-SFPRA	0.819	0.689–0.898
AR-SFPA	0.772	0.616–0.869
R-SFSRA	0.864	0.763–0.924
R-SFIRA	0.778	0.626–0.873
CR-SFPA	0.853	0.744–0.917

CI: confidence interval. L: left, R: right, SFARA: Sylvian fissure anterior rotation angle, SFPRA: Sylvian fissure posterior rotation angle, SFSRA: Sylvian fissure superior rotation angle, SFIRA: Sylvian fissure inferior rotation angle, AL-SFPA: left axial Sylvian fissure plateau angle, AR-SFPA: right axial Sylvian fissure plateau angle, CL-SFPA: left coronal Sylvian fissure plateau angle, CR-SFPA: right coronal Sylvian fissure plateau angle.

### 3.6 An example of predictive model validation

**Figure 4:**
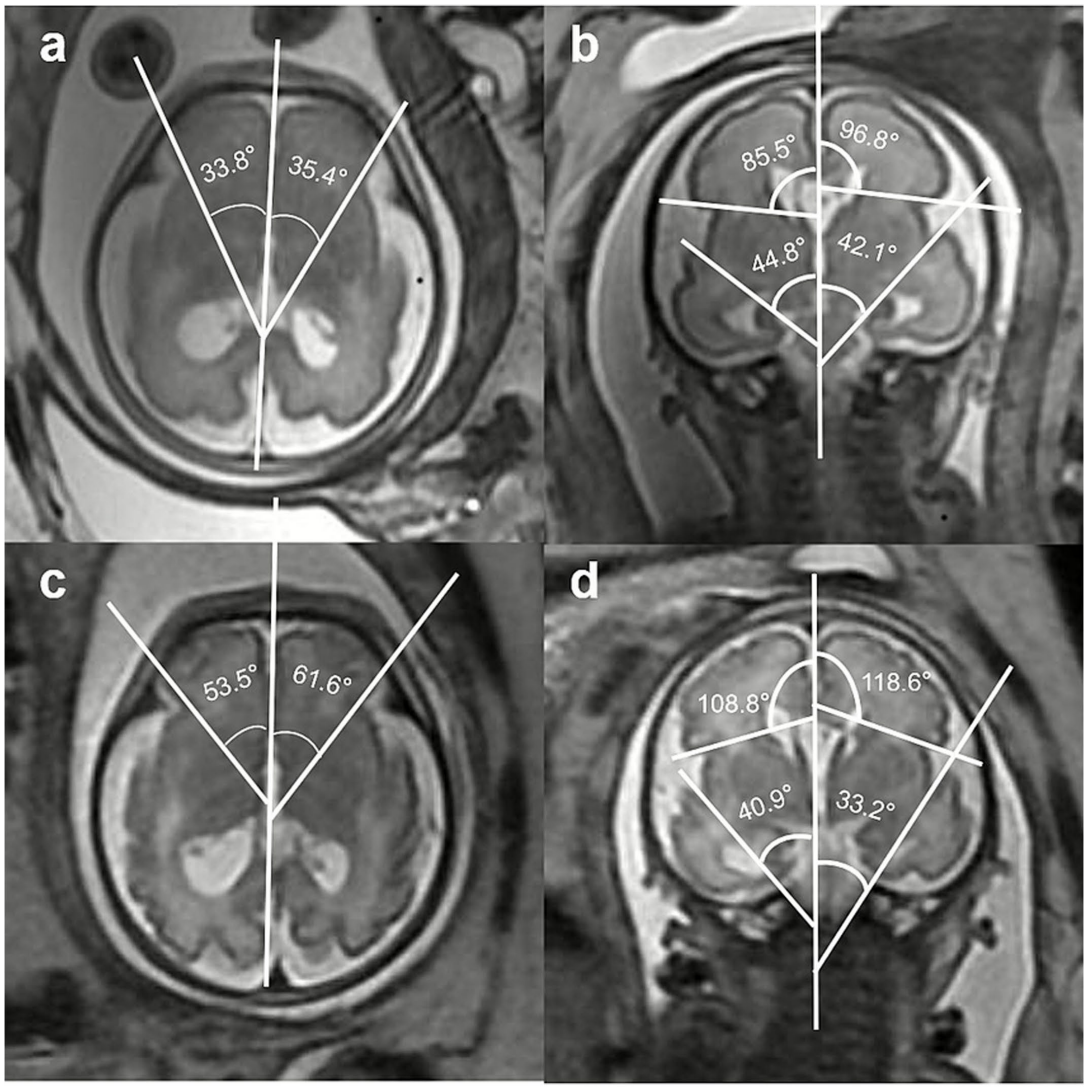
Fetus with cortical dysplasia presenting with abnormal Sylvian fissure angles (SFAs). **(a,b)** Sequentially show the angle measurements of the bilateral Sylvian fissure anterior rotation angle (SFARA) in the axial view and the bilateral Sylvian fissure inferior rotation angle (SFIRA) and the Sylvian fissure superior rotation angle (SFSRA) in the coronal view at 28 + 2 weeks of gestation. **(c,d)** Sequentially show the angle measurements of the bilateral SFARA in the axial view and the bilateral SFIRA and the SFSRA in the coronal view at 32 weeks of gestation.

**Figure 5:**
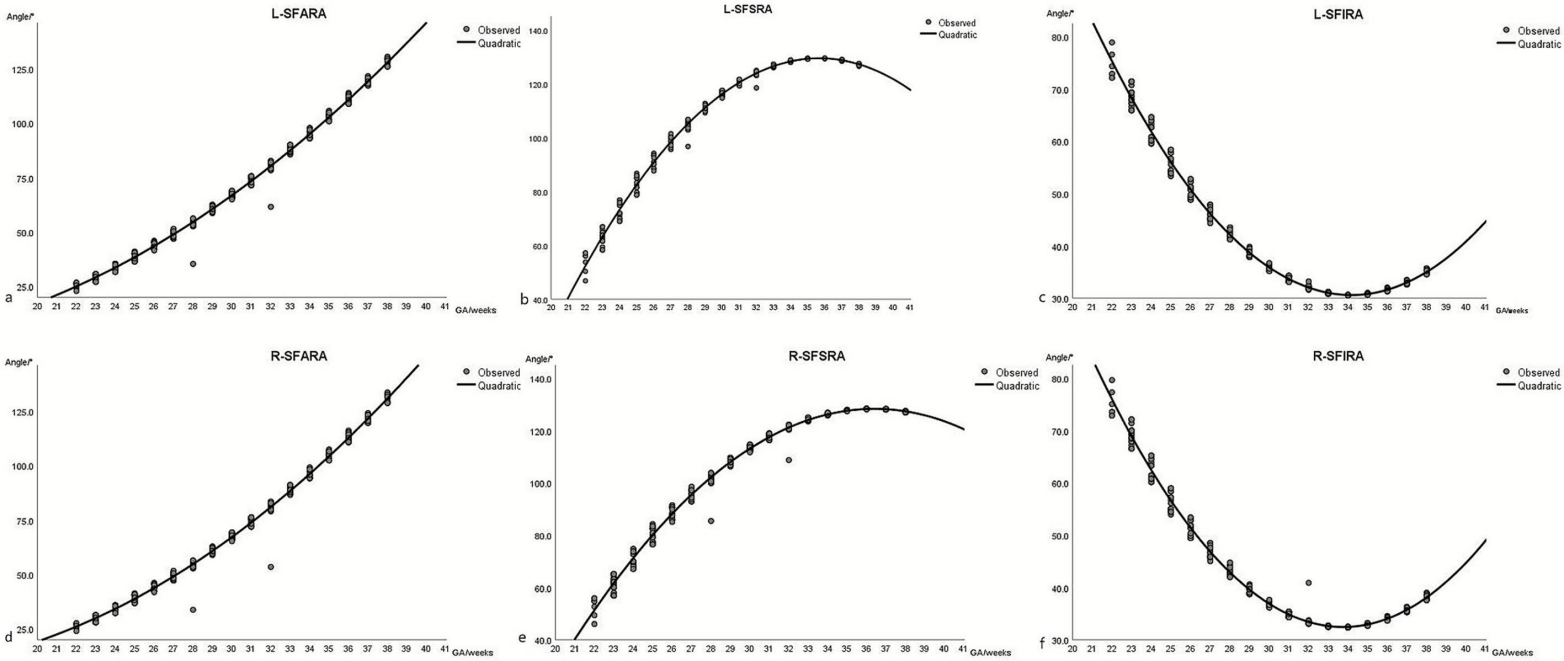
Fetus with cortical dysplasia: abnormal Sylvian fissure angle (SFA) identification aided by predictive model curves. GA: gestational age, L: left, R: right, SFARA: Sylvian fissure anterior rotation angle, SFSRA: Sylvian fissure superior rotation angle, SFIRA: Sylvian fissure inferior rotation angle. Panels **a–c** sequentially show the left-sided SFARA, SFSRA, and SFIRA angles of a MCD fetus, all outside the prediction model curves; panels **d–f** display the corresponding right-sided angles against the prediction model curves.

Measurement values of the SFAs of the fetus with MCD are shown in Figure 4. At 28 + 2 weeks of gestation (Figures 4a,b), the R-SFARA was 33.8° < 52.9° (5th percentile), the L-SFARA was 35.4° < 52.7° (5th percentile), the R-SFSRA was 85.5° < 102° (5th percentile), the L-SFSRA was 96.8° < 104.9° (5th percentile), the R-SFIRA was 44.8° > 44.4° (95th percentile), and the L-SFIRA was 42.1° (5th–95th percentiles). At 32 weeks of gestation (Figures 4c,d), the R-SFARA was 53.5° < 79.1° (5th percentile), the L-SFARA was 61.6° < 78.5° (5th percentile), the R-SFSRA was 108.8° < 120.4° (5th percentile), the L-SFSRA was 118.6° < 123.2° (5th percentile), the R-SFIRA was 40.9° > 33.7° (95th percentile), and the L-SFIRA was 33.2° > 32.4° (95th percentile). Figures 5a–f demonstrates that the abnormal SFA measurements of this MCD fetus lie outside the fitted curves.

## 4 Discussion

This study revealed that Sylvian fissure angles change with gestational age during normal fetal development, following a predictable trajectory that can be modeled mathematically. Then, bilateral differences were observed in the majority of SFAs. The assessment of the SFAs has good intra-rater and inter-rater reliability. Our study established the reference range and the development model of Sylvian fissure angles, which laid a foundation for the early detection of MCD.

Several studies on the Sylvian fissure have been published. The majority of them focus on measuring its length or depth, while others examine parameters such as angle, area, or perimeter. Zeng et al. (2023) prospectively measured the Sylvian fissure plateau angle of 180 normal fetuses using ultrasound and proposed the concept of “Sylvian fissure plateau angle” to predict MCD. While the Sylvian fissure plateau angle is an important parameter during the development of the Sylvian fissure, it remains stable throughout pregnancy. Therefore, we investigated additional angles in addition to the Sylvian fissure plateau angle.

Quarello et al. (2008) defined the progressive overriding of the insula by the temporal lobe on a scale of 0 to 10 through a scoring sheet and a semiquantitative assessment. Although they provide a simple and reproducible method to evaluate Sylvian fissure operculization, the approach introduces some degree of subjective influence. Liat et al. measured the area and perimeter of the Sylvian fissure in 55 fetuses prospectively to demonstrate a linear growth along gestation. While the sample size was modest, the prospective design enhances the validity of temporal growth observations (Gindes et al., 2015). Poon et al. (2019) prospectively studied the angle of the Sylvian fissure in 422 fetuses to develop reference ranges of Sylvian fissure angles during normal pregnancy at 18–30 weeks of gestation, and they confirmed that the Sylvian fissure angle was increased in fetuses with MCD (Pooh et al., 2019). Their study demonstrated two principal strengths: a prospective cross-sectional design and a substantial sample size. However, we contended that their chosen horizontal reference line introduces measurement complexity by generating negative angle values in the results. In contrast, our methodology used a vertical reference line to eliminate this issue and maintain positive angular measurements throughout.

Gabriela et al. proved that there are differences in cortical development between late-onset intrauterine growth restricted (IUGR) fetuses and normally grown fetuses (Egana-Ugrinovic et al., 2013). They evaluated cortical development parameters by MRI, but their study was limited to fetuses at 37 weeks of gestation, lacking longitudinal continuity. Our study measured Sylvian fissure angles in fetuses at 22–38 weeks of gestation and established reference ranges, providing a theoretical basis for future investigations of factors influencing cortical development during pregnancy.

De Vareilles et al. (2023) found that the development of the Sylvian fissure was asymmetrical, which coincided with our study. Lateral differences in angular measurements were statistically significant (*p <* 0.05) for the majority of parameters except for the SFARA (*p* = 0.347). Further investigation is warranted to elucidate the underlying anatomical and functional implications of these asymmetries.

In our study, although there was no statistically significant difference in the SFARA between the right and left Sylvian fissures, the SFPRA and the SFIRA were significantly smaller than those on the right side, while the SFSRA was significantly larger than that on the right side. These findings are sufficient to indicate that the development of the left Sylvian fissure is more mature than that of the right side, which is consistent with previous studies reporting anatomical asymmetry in Sylvian fissure development. The development of the asymmetric Sylvian fissure involves three key mechanisms. Genetically, differential gene expression in opercular/insular cortices and the 14q23.1 locus regulates it (Mallela et al., 2020). Anatomically, the left hemisphere’s more pronounced opercularization and growth differences cause a longer, flatter left fissure (de Vareilles et al., 2022). Functionally, language lateralization shapes left perisylvian structures (Sprung-Much et al., 2022).

Our research model successfully identified a fetus with MCD at 28 + 2 weeks of gestation. Both the bilateral SFARA and SFSRA of this fetus were below the 5th percentile, while the SFIRA exceeded the 95th percentile. These abnormalities persisted during the reexamination at 32 weeks of gestation. Although the left SFIRA fell within the range of the 5th to 95th percentiles at 28 + 2 weeks of gestation, it exceeded the 95th percentile during the follow-up reexamination at 32 weeks. Finally, whole-exome sequencing (WES) of amniotic fluid identified a heterozygous variant in the PEX1 gene, which showed a good correlation with the MRI findings. This result confirms the efficacy and reliability of our model in detecting fetal MCD.

### 4.1 Limitations

The study has several limitations. First, our data were collected retrospectively from a single-center cohort. As fetal MRI represents a targeted rather than routine examination modality, prospective case collection remains challenging in clinical practice. Second, the insufficient sample size of fetuses at extreme gestational ages (22 weeks and 38 weeks) leads to inadequate representativeness of the sample and may undermine the accuracy of the centile curves for these specific gestational ages. Third, the number of verified cases is small. To address this, subsequent research will involve retrospective collection of a larger cohort of fetuses with MCD for model validation, coupled with prospective application of the model in the detection of fetal cortical developmental malformations. Additionally, future investigations should incorporate multicenter, large-sample datasets to further corroborate our findings.

## 5 Conclusion

We have proposed the concept of the rotation angle for the first time. The term “rotation” well reflects a process of the dynamic development of the Sylvian fissure. We have verified the growth pattern of Sylvian fissure angles as they change with gestation, and the measurement has good stability and repeatability, which can provide a solid theoretical basis for the early diagnosis of cortical developmental malformations by MRI in the future.

## Data Availability

The raw data supporting the conclusions of this article will be made available by the authors, without undue reservation.
