# Supplementary material for: Sylvian fissure angle development on fetal MRI: 22–38 gestational weeks
Source: Front Neurosci. 2026 Jan 5;19:1686398. doi: 10.3389/fnins.2025.1686398 (PMC12813082; doi:10.3389/fnins.2025.1686398)
Supplement: Supplementary file 1 [file Table_1.DOCX]

Table S1. Median and Quartile Interval of Fetal Axial Sylvian Fissure Angles Measurements for Gestational Age in Weeks Rounded Down.

|  |  | L-SFARA | | | R-SFARA | | | L-SFPRA | | | R-SFPRA | | | AL-SFPA | | | AR-SFPA | | |
| --- | --- | --- | --- | --- | --- | --- | --- | --- | --- | --- | --- | --- | --- | --- | --- | --- | --- | --- | --- |
| GA | N | Median | 25th | 75th | Median | 25th | 75th | Median | 25th | 75th | Median | 25th | 75th | Median | 25th | 75th | Median | 25th | 75th |
| total | 324 | 62.3 | 41.3 | 103.9 | 58.4 | 41.9 | 103.6 | 26.2 | 18.5 | 36.3 | 28.3 | 20.6 | 40.1 | 14.5 | 10.7 | 18.9 | 13.6 | 9.6 | 18.0 |
| 22 | 8 | 31.9 | 28.2 | 37.4 | 32.3 | 29.4 | 37.7 | 98.3 | 48.2 | 112.1 | 104.1 | 48.1 | 109.9 | 19.2 | 16.4 | 24.6 | 25.1 | 18.5 | 26.0 |
| 23 | 24 | 31.4 | 27.1 | 37.7 | 29.5 | 22.8 | 36.5 | 56.0 | 37.5 | 86.6 | 63.9 | 45.5 | 108.2 | 20.3 | 17.3 | 23.7 | 16.8 | 11.3 | 20.7 |
| 24 | 21 | 34.5 | 30.1 | 37.9 | 36.9 | 31.4 | 40.9 | 47.4 | 32.4 | 98.8 | 55.7 | 43.0 | 99.2 | 23.1 | 18.8 | 28.8 | 17.0 | 14.5 | 24.3 |
| 25 | 20 | 32.6 | 28.5 | 38.7 | 36.1 | 32.4 | 44.3 | 29.5 | 24.3 | 42.9 | 48.6 | 35.2 | 85.0 | 18.3 | 15.2 | 23.2 | 19.1 | 14.4 | 23.9 |
| 26 | 18 | 36.9 | 32.7 | 44.6 | 41.9 | 38.2 | 47.8 | 36.6 | 25.8 | 48.8 | 43.1 | 30.8 | 68.1 | 24.1 | 15.4 | 27.1 | 19.8 | 16.5 | 27.2 |
| 27 | 19 | 47.2 | 42.3 | 52.2 | 50.4 | 39.8 | 54.3 | 29.0 | 21.9 | 33.9 | 27.9 | 25.0 | 33.5 | 13.5 | 11.6 | 17.1 | 12.0 | 9.5 | 14.7 |
| 28 | 19 | 54.1 | 48.4 | 67.9 | 52.9 | 44.4 | 71.5 | 24.6 | 19.6 | 30.1 | 21.4 | 14.9 | 27.5 | 16.1 | 11.3 | 19.4 | 12.1 | 10.1 | 17.0 |
| 29 | 19 | 56.5 | 49.1 | 79.1 | 51.5 | 47.7 | 57.3 | 19.7 | 16.8 | 25.2 | 17.1 | 11.4 | 29.5 | 14.6 | 12.1 | 18.9 | 14.9 | 10.9 | 20.2 |
| 30 | 25 | 59.3 | 51.5 | 74.1 | 56.0 | 52.5 | 73.3 | 23.9 | 19.1 | 31.8 | 23.3 | 15.9 | 37.1 | 11.8 | 8.1 | 15.2 | 11.6 | 7.9 | 15.5 |
| 31 | 20 | 61.9 | 51.5 | 70.2 | 66.7 | 53.5 | 91.0 | 24.2 | 19.7 | 34.9 | 26.3 | 21.1 | 28.7 | 13.6 | 8.6 | 17.3 | 13.0 | 9.9 | 16.3 |
| 32 | 20 | 82.7 | 62.0 | 96.8 | 84.0 | 74.6 | 95.8 | 24.7 | 18.8 | 27.5 | 30.3 | 21.8 | 39.6 | 15.1 | 13.2 | 17.7 | 14.6 | 9.7 | 17.0 |
| 33 | 20 | 98.9 | 92.5 | 105.4 | 91.3 | 87.7 | 101.3 | 28.1 | 19.8 | 36.2 | 28.3 | 24.8 | 37.1 | 14.0 | 10.5 | 17.3 | 15.4 | 11.7 | 17.6 |
| 34 | 18 | 103.8 | 94.8 | 119.3 | 98.1 | 94.0 | 107.4 | 28.5 | 20.9 | 33.9 | 29.3 | 22.2 | 32.3 | 13.6 | 9.8 | 16.3 | 11.4 | 7.9 | 15.9 |
| 35 | 21 | 111.0 | 106.7 | 119.3 | 117.2 | 111.2 | 123.2 | 19.3 | 15.2 | 27.3 | 20.0 | 14.7 | 23.8 | 11.0 | 8.8 | 13.7 | 9.8 | 7.0 | 12.3 |
| 36 | 20 | 113.9 | 107.0 | 120.1 | 119.3 | 112.7 | 126.9 | 18.0 | 13.6 | 25.4 | 20.1 | 15.5 | 25.3 | 10.2 | 7.8 | 14.7 | 8.0 | 6.1 | 12.0 |
| 37 | 20 | 114.6 | 111.1 | 121.4 | 117.3 | 110.5 | 121.3 | 17.3 | 11.7 | 24.2 | 23.0 | 15.5 | 26.8 | 11.2 | 8.2 | 13.6 | 10.3 | 8.6 | 15.0 |
| 38 | 12 | 112.5 | 106.1 | 116.6 | 114.8 | 111.7 | 119.5 | 18.2 | 16.1 | 32.2 | 18.8 | 14.2 | 23.7 | 9.7 | 6.9 | 11.2 | 10.4 | 8.4 | 16.7 |

L: left, R: right, SFARA: Sylvian fissure anterior rotation angle, SFPRA: Sylvian fissure posterior rotation angle, AL-SFPA: Left axial Sylvian fissure plateau angle, AR-SFPA: Right axial Sylvian fissure plateau angle.

Table S2. Median and Quartile Interval of Fetal Coronal Sylvian Fissure Angles Measurements for Gestational Age in Weeks Rounded Down.

|  |  | L-SFSRA | | | R-SFSRA | | | L-SFIRA | | | R-SFIRA | | | CL-SFPA | | | CR-SFPA | | |
| --- | --- | --- | --- | --- | --- | --- | --- | --- | --- | --- | --- | --- | --- | --- | --- | --- | --- | --- | --- |
| GA | N | Median | 25th | 75th | Median | 25th | 75th | Median | 25th | 75th | Median | 25th | 75th | Median | 25th | 75th | Median | 25th | 75th |
| total | 324 | 115.7 | 99.3 | 126.7 | 111.9 | 93.4 | 125.7 | 37.7 | 29.7 | 50.1 | 38.6 | 32.1 | 51.2 | 10.3 | 6.9 | 13.7 | 11.1 | 7.8 | 14.1 |
| 22 | 8 | 52.3 | 47.1 | 57.9 | 53.3 | 44.8 | 64.5 | 67.0 | 53.8 | 70.0 | 69.9 | 64.5 | 87.5 | 11.1 | 9.4 | 14.5 | 12.3 | 9.2 | 14.4 |
| 23 | 24 | 53.9 | 45.2 | 61.5 | 50.7 | 46.7 | 61.6 | 70.7 | 59.1 | 92.3 | 74.6 | 54.5 | 95.0 | 10.7 | 7.7 | 13.9 | 12.4 | 7.4 | 16.1 |
| 24 | 21 | 67.8 | 62.2 | 75.8 | 63.3 | 53.0 | 72.8 | 69.1 | 59.4 | 76.8 | 58.4 | 44.7 | 79.8 | 11.0 | 6.8 | 13.1 | 11.1 | 9.6 | 13.4 |
| 25 | 20 | 78.8 | 56.3 | 94.0 | 78.1 | 58.1 | 91.7 | 58.6 | 44.4 | 64.8 | 60.3 | 46.9 | 66.1 | 9.3 | 5.6 | 11.9 | 10.7 | 9.3 | 12.3 |
| 26 | 18 | 101.4 | 91.4 | 109.6 | 98.5 | 94.6 | 105.7 | 49.6 | 42.7 | 59.2 | 51.4 | 46.5 | 57.8 | 13.0 | 9.0 | 16.4 | 11.5 | 9.2 | 14.8 |
| 27 | 19 | 108.4 | 103.0 | 116.6 | 106.9 | 97.6 | 117.1 | 35.8 | 28.4 | 41.4 | 36.2 | 31.2 | 41.8 | 13.9 | 10.7 | 19.1 | 15.5 | 12.3 | 18.5 |
| 28 | 19 | 111.8 | 102.7 | 117.8 | 110.9 | 105.7 | 122.5 | 34.2 | 26.4 | 40.8 | 34.1 | 27.2 | 46.8 | 13.0 | 9.0 | 17.6 | 11.7 | 7.8 | 17.6 |
| 29 | 19 | 116.3 | 114.0 | 120.0 | 113.2 | 111.0 | 119.0 | 32.7 | 23.1 | 34.8 | 34.0 | 21.2 | 37.4 | 13.8 | 10.3 | 15.9 | 12.9 | 10.6 | 17.7 |
| 30 | 25 | 116.2 | 111.9 | 124.2 | 116.5 | 108.2 | 123.0 | 33.8 | 26.7 | 39.2 | 33.0 | 24.2 | 46.3 | 7.4 | 4.5 | 12.2 | 8.7 | 6.2 | 13.1 |
| 31 | 20 | 108.5 | 106.2 | 120.3 | 111.8 | 108.6 | 117.6 | 36.7 | 34.4 | 40.1 | 41.9 | 38.9 | 47.8 | 7.5 | 5.1 | 9.9 | 7.2 | 4.8 | 10.5 |
| 32 | 20 | 118.8 | 116.0 | 121.1 | 110.5 | 105.1 | 118.3 | 37.9 | 33.2 | 40.4 | 36.7 | 31.6 | 39.4 | 10.6 | 5.5 | 11.4 | 12.0 | 7.2 | 14.9 |
| 33 | 20 | 120.5 | 113.2 | 126.9 | 115.7 | 107.1 | 120.8 | 37.5 | 32.4 | 45.0 | 36.9 | 33.2 | 50.0 | 12.0 | 9.8 | 15.6 | 12.1 | 9.4 | 15.5 |
| 34 | 18 | 126.2 | 119.6 | 129.4 | 122.6 | 116.1 | 126.1 | 40.7 | 35.9 | 44.2 | 40.2 | 33.3 | 47.7 | 6.2 | 4.5 | 8.9 | 6.5 | 4.5 | 9.4 |
| 35 | 21 | 131.4 | 127.9 | 134.8 | 130.9 | 128.5 | 134.1 | 29.3 | 24.2 | 34.9 | 32.3 | 25.7 | 38.1 | 9.2 | 6.4 | 12.3 | 11.8 | 8.8 | 13.9 |
| 36 | 20 | 132.0 | 126.7 | 136.6 | 131.8 | 127.5 | 134.3 | 28.0 | 24.2 | 35.4 | 32.4 | 26.1 | 35.9 | 10.0 | 7.9 | 13.9 | 11.7 | 10.2 | 13.5 |
| 37 | 20 | 133.1 | 126.0 | 135.5 | 130.0 | 126.9 | 137.5 | 29.5 | 26.2 | 32.3 | 32.8 | 29.3 | 37.9 | 9.1 | 7.6 | 13.3 | 10.2 | 6.6 | 12.0 |
| 38 | 12 | 132.0 | 129.0 | 135.3 | 135.5 | 128.4 | 140.1 | 29.4 | 19.8 | 37.2 | 30.3 | 27.0 | 40.1 | 8.3 | 7.5 | 11.4 | 8.6 | 6.1 | 11.0 |

L: left, R: right, SFSRA: Sylvian fissure superior rotation angle, SFIRA: Sylvian fissure inferior rotation angle, CL-SFPA: Left coronal Sylvian fissure plateau angle, CR-SFPA: Right coronal Sylvian fissure plateau angle.
